# Deforestation and Vectorial Capacity of *Anopheles gambiae* Giles Mosquitoes in Malaria Transmission, Kenya 

**DOI:** 10.3201/eid1410.070781

**Published:** 2008-10

**Authors:** Yaw A. Afrane, Tom J. Little, Bernard W. Lawson, Andrew K. Githeko, Guiyun Yan

**Affiliations:** Kenya Medical Research Institute, Kisumu, Kenya (Y.A. Afrane, A.K. Githeko); University of Edinburgh, Edinburgh, Scotland, UK (T.J. Little); Kwame Nkrumah University of Science and Technology, Kumasi, Ghana (Y.A. Afrane, B.W. Lawson); University of California, Irvine, California, USA (G. Yan)

**Keywords:** Mosquitoes, malaria, deforestation, vectors, Anopheles gambiae, climate, Kenya, research

## Abstract

We investigated the effects of deforestation on microclimates and sporogonic development of *Plasmodium falciparum* parasites in *Anopheles gambiae* mosquitoes in an area of the western Kenyan highland prone to malaria epidemics. *An*. *gambiae* mosquitoes were fed with *P. falciparum*–infected blood through membrane feeders. Fed mosquitoes were placed in houses in forested and deforested areas in a highland area (1,500 m above sea level) and monitored for parasite development. Deforested sites had higher temperatures and relative humidities, and the overall infection rate of mosquitoes was increased compared with that in forested sites. Sporozoites appeared on average 1.1 days earlier in deforested areas. Vectorial capacity was estimated to be 77.7% higher in the deforested site than in the forested site. We showed that deforestation changes microclimates, leading to more rapid sporogonic development of *P. falciparum* and to a marked increase of malaria risk in the western Kenyan highland.

## Abstract 2

One-sentence summary for table of contents: Land use changes are affecting malaria transmission in this region.

Land use changes in the form of deforestation and swamp cultivation have been occurring rapidly in the western Kenyan highlands because of unprecedented human demand for forest products and land for agricultural cultivation (*1*,*2*). In the East African highlands, 2.9 million hectares of forest were cleared between 1981 and 1990, representing an 8% reduction in forest cover in 1 decade (*3*). As has been observed in the Usambara Mountains of Tanzania (*4*) and in the southwestern highlands of Uganda (*5*), land use change in Kenya may have exacerbated malaria epidemics caused by *Plasmodium falciparum* parasites and its mosquito vectors *Anopheles gambiae* and *An*. *funestus* (*6*–*9*), although other factors may have also contributed to the surge in epidemics, including global warming (*10*,*11*), climate variability (*12*), and drug resistance (*4*,*13*). Land use change can influence malaria transmission by increasing the temperature and decreasing the humidity of vector mosquito habitats. This in turn affects biting, survival, and reproductive rates of vectors (*8*,*9*,*14*,*15*).

Temperature changes will also shorten the development time of *P*. *falciparum* in mosquitoes (*16*–*18*). Development of malaria parasites in mosquitoes (sporogony) involves a sequential process of developmental steps. Gametocytes ingested by anopheline mosquitoes develop into zygotes, ookinetes, oocysts, and eventually sporozoites in the salivary glands, ready for transmission to the next human host. Generally, the extrinsic incubation period of *P*. *falciparum* is inversely correlated with temperature. At 27°C, the extrinsic incubation period of *P*. *falciparum* from zygotes to sporozoites was found to be 12 days (*19*,*20*). The relationship between temperature and extrinsic incubation period is not linear because small changes at low temperatures can have proportionally large effects on parasites’ extrinsic incubation period. Thus, geographic distribution of malaria is confined within climates favoring its extrinsic cycle, provided that other conditions do not limit mosquito survival (*21*). In turn, duration of the sporogonic development of malaria parasites in mosquitoes is an important component of vectorial capacity. Vectorial capacity measures the potential rate of contact between infectious vectors and susceptible hosts.

The objective of this study was to assess the effect of deforestation on the microclimate (temperature and humidity) of houses where mosquitoes normally reside with their parasites and to investigate how these microclimate changes affect the sporogonic development of the malaria parasite within *An*. *gambiae*. Humidity may not have a direct effect on the sporogonic development of malaria parasites, but it may affect mosquito survival through the combined effect of temperature and humidity (saturation deficit). We hypothesized that land use changes in the highlands alter the microclimatic conditions of vector mosquitoes and their parasites, subsequently enhancing malaria risk in the area.

## Materials and Methods

### Study Sites

This study was conducted at a highland site in Iguhu village (34°45′E, 0°10′N, 1,430–1,580 m above sea level) in Kakamega district in western Kenya. A lowland site in Kisian village (34°75′E, 0°10′S, 1,190 m above sea level) in Kisumu district in western Kenya was studied for comparison. The highland area of Kakamega district has 2 rainy seasons and ≈1,800 mm of rainfall per year. The long rainy season usually occurs between mid-March and May, with an average monthly rainfall 150–260 mm, and the short rainy season usually occurs between September and October, with an average monthly rainfall of 165 mm. Malaria prevalence peaks usually lag 1–2 months after the rain (*22*). The mean annual daily temperature is 20.8°C. A natural forest is located east of the study area, constituting ≈15% of the total area. The rest of the area is a continuum of forest in different states of deforestation. A forested area was defined as an area having a tree canopy cover >60%. The deforested area has canopy cover <10%. The deforested area was once forested but the forest has been cut down. The predominant malaria vector species in the area is *An*. *gambiae* s.s (*6*,*23*).

We also studied 1 lowland site (Kisian) as a comparison site for parasite development. Kisian (1,200 m above sea level) is flat land located on the shores of Lake Victoria where malaria transmission is perennial and malaria vectors include *An*. *gambiae* s. s., *An*. *arabiensis*, and *An*. *funestus* (*24*,*25*). The land cover type in Kisian is primarily farmland with little tree canopy coverage; thus, Kisian is classified as a deforested area. The average minimum and maximum temperatures during 1970–2000 were 15.0°C and 28.4°C, respectively. The average annual rainfall within this same period was ≈1,400 mm.

### Selection of *P*. *falciparum* Gametocyte Carriers

The study population was primary school students in Iguhu. Children 5–14 years of age were recruited for this study and were screened monthly for *P*. *falciparum* gametocytes in their blood. The ethical review boards of the Kenya Medical Research Institute, Kenya, and the University of California, Irvine, reviewed and approved the protocol for screening of *P*. *falciparum* gametocyte carriers and subsequent mosquito infections. Parents or guardians, as well as the children involved in the study, signed a consent or assent form to participate in this study.

### Mosquito Infection

Students (gametocyte carriers) who had >40 gametocytes/μL of blood and who consented to participate in the study were asked to donate 10 mL of blood, which was obtained by a clinician. Butterfly needles were used in drawing the blood into a heparinized tube. This blood was immediately centrifuged at 2,000 rpm for 5 min. The supernatant (serum) was discarded and replaced with human AB serum (Cambrex Bio Science, Walkersville, MD, USA). All equipment used was warmed to 37°C. After replacement of the serum, the blood was placed in warmed artificial membrane feeders. *An*. *gambiae* mosquitoes were placed in paper cups at a density of 60/cup and allowed to feed on the infected blood for 30 min. Fed mosquitoes were divided and placed in 3 cages (30 cm^3^), which were hung in bedrooms of houses in deforested and forested areas in Iguhu and in the lowland site. Four houses were used at each site. At each mosquito feeding, 600 mosquitoes were provided an opportunity to blood feed and an average of 60–80% of mosquitoes took a blood meal. Fed mosquitoes were distributed equally in cages at the 3 sites. *An*. *gambiae* mosquitoes used in this experiment were obtained from the village of Iguhu but had been bred in an insectary and adapted to feed from a membrane feeder. All gametocyte donors were treated with amodiaquine by the clinicians in Iguhu Health Centre. Feeding of mosquitoes was conducted in a secure, insect-proof room at the Iguhu Health Centre. Fed mosquitoes were transported to different sites in paper cups placed in cool boxes.

### Dissection for Oocysts and Sporozoites

Starting at 5 days after mosquitoes were exposed to different house environments, 30 mosquitoes from each cage at each site were dissected daily in 2% mercurochrome and examined for oocysts. The gut of each mosquito was carefully drawn out of the abdomen and observed under a light microscope. Infected mosquitoes were counted and the number was recorded; the oocyst load was expressed as number of oocysts per infected mosquito. Dissection for sporozoites started on day 9 postinfection. Salivary glands of each mosquito were taken from the thorax and crushed under a coverslip. This material was then observed under a microscope for sporozoites. This part of the study was conducted during April–November 2005.

### Climate Data Collection

HOBO data loggers (Onset Computer Corporation, Bourne, MA, USA), which are devices for measuring temperature and humidity, were placed inside all experimental houses where the duration of sporogony of *P*. *falciparum* was measured to record temperature and relative humidity hourly. Data loggers were suspended from the roof 2 m above the ground. Outdoor temperature was recorded by using these data loggers placed in standard meteorologic boxes 2 m above the ground. All selected houses had a data logger for indoor and outdoor microclimate measurements. These data were offloaded from the data loggers by using a HOBO shuttle data transporter (Shuttle; Onset Computer Corporation) and downloaded to a computer by using BoxCar Pro version 4.0 (Onset Computer Corporation).

### Statistical Analysis

Daily mean maximum, mean, and minimum temperatures were calculated from hourly temperature and humidity data recorded throughout the experimental period. Analysis of variance with repeated measures was used to compare monthly mean (based on the average per house in each site) temperatures throughout the entire study period. Analysis of variance was also used to test the effect of site (a fixed effect with 3 levels: forested, deforested, or lowland) and donor child (a random effect) on the average (per cage) time to sporozoite detection, the average (per cage) oocyst load, or the infection rate. Time to sporozoite infection was defined as the time postinfection needed for sporozoites to appear in dissected mosquitoes. Infection rate was defined as the proportion of mosquitoes containing oocysts or sporozoites per total number of mosquitoes dissected in each cage. This proportion was arcsin square root transformed before analysis. Oocyst load was defined as the average of oocyst counts in all the positive mosquito midguts. A total of 34 membrane feedings (34 gametocyte donors) were conducted; 29 feedings yielded infection rates >10% and were used in the data analysis. Analyses were performed with the JMP statistical package (*26*).

To evaluate how the time for sporozoite development affects vectorial capacity, we used the formula of MacDonald (*17*). For this analysis, vectorial capacity = *ma*^2^*p*^n^/(–ln(*p*)), in which *m* is the relative density of vectors in relation to human density, *a* is the average number of persons bitten by 1 mosquito in 1 day, *p* is the proportion of vectors surviving per day, and n is the duration of sporogony in days. Therefore, vectorial capacity is the number of future infections that will arise from 1 current infective case. Vector abundance was calculated from the population dynamics data from the study area during June 2003–June 2004 (*27*), which was stratified by land use type. Parameter *a* was calculated as 2/first gonotrophic cycle duration that we estimated from the same study site (*8*), which was also stratified by land use type, assuming that double feeding is required for 1 gonotrophic cycle. Parameter *p* was estimated from results of our previous study on adult survival in the same setting, which is an approximation to observed age-dependent survival (*9*). Duration of sporogony was calculated on the basis of data from the present study.

## Results

### Indoor Environment

**Figure 1:**
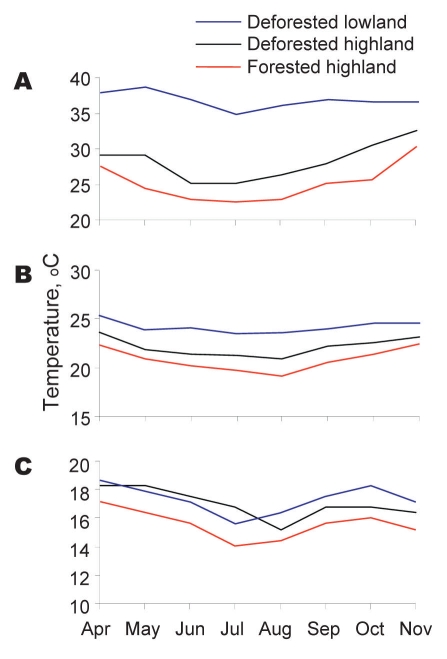
Monthly average value of daily maximum (A), mean (B), and minimum (C) indoor temperatures in forested and deforested areas in western Kenyan highland (Kakamega) and deforested lowland (Kisian), April–November 2005.

Mean indoor temperatures in experimental houses during the entire experimental period differed significantly among the 3 sites (forested area 21.5°C, deforested area 22.4°C, lowland 23.9°C; F_2,8_ = 4.5, p<0.05) (Figure 1). Post hoc contrast showed a difference between the lowland and either highland site. Indoor maximum and minimum temperatures mirrored this difference. Mean indoor humidity within experimental houses also differed significantly among the 3 sites (forested area 75.9%, deforested area 60.6%, lowland 64.7%; F_2,8_ = 8.1, p<0.05) (Table 1). Post hoc contrast showed a significant difference between the lowland and both highland sites and between the forested and deforested areas in the highland (F_1,7_ = 14.5, p<0.001).

**Table 1:** Climatic conditions and *Plasmodium falciparum* infection in *Anopheles gambiae* mosquitoes fed infected blood meals according to site and land use type, western Kenyan highland and lowland*

Site	Land use type	Mean ± SD indoor temperature, °C	Mean ± SD indoor relative humidity, %	No. membrane feedings	No. feedings resulting in infection	Total no. mosquitoes dissected	Range of infection rates,† %
Highland	Forested	20.89 ± 0.39^a^	75.9 ± 1.8^a^	34	27	3,171	10.0–42.8
Highland	Deforested	22.13 ± 0.27^b^	60.6 ± 2.1^b^	34	29	3,719	10.2–40.6
Lowland	Deforested	23.90 ± 0.40^c^	64.7 ± 2.1^b^	21	19	1,749	11.0–42.4

*Superscript letters after values indicate results of Tukey-type multiple comparison tests. Values with the same superscript letter in the same column were not statistically significant (p = 0.05). †Infection rate refers to proportion of dissected mosquitoes that were infected with *P. falciparum* oocysts or sporozoites.

### Outdoor Environment

Mean outdoor temperatures within the experimental houses during the entire experimental period differed significantly among the 3 sites (forested 19.0°C, deforested 19.9°C, lowland 22.4°C; F_2,8_ = 58.1, p<0.0001). Post hoc contrast showed a significant difference between the lowland and either highland site, and also between the forested and deforested areas in the highland (F_1,8_ = 82.8, p<0.05). Indoor maximum and minimum temperatures mirrored this difference. Mean outdoor humidity did not differ among the 3 sites (F_2,8_ = 2.6, p>0.05); this was true also for maximum and minimum humidity values.

### Parasite Infection and Development

**Figure 2:**
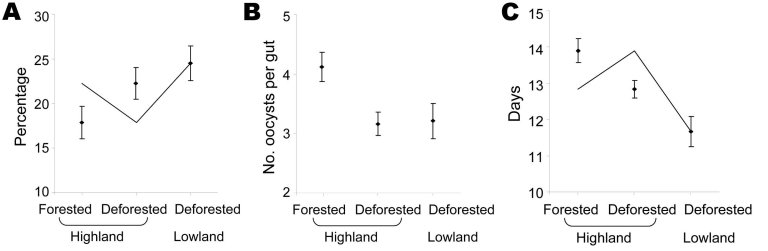
Mean infection rate (A), mean oocyst intensity (B), and time to sporozoite detection (C) in forested and deforested areas in western Kenyan highland (Kakamega) and deforested lowland (Kisian), April–November 2005. Error bars represent standard error.

The number of oocysts in a mosquito ranged from 1 to 98. The proportion of mosquitoes that carried *P*. *falciparum* infection (either oocysts or sporozoites) differed significantly among the sites (F_2,30_ = 12.1, p<0.0001) (Figure 2, panel **A**), and post hoc contrast indicated a significant forested–deforested difference within the highland site (F_1,30_ = 16.5, p<0.0001). Mean oocyst intensity was affected by land use type (F_2,30_ = 6.5, p<0.01) (Figure 2, panel **B**). Time for sporozoite development differed between sites (F_2,13_ = 9.1, p<0.01), and post hoc contrasts indicated a significant difference between forested and deforested areas in the highland site (F_1,13_ = 6.9, p<0.05) (Figure 2, panel **C**), and the lowland site exhibited the shortest sporozoite development time. Post hoc contrasts did not result in different conclusions from the alternative approach of reducing the number of factor levels in the dataset (e.g., comparing forested with deforested sites in the absence of lowland data).

### Effects of Deforestation on Vectorial Capacity

Vectorial capacity at the 3 study sites was estimated by using the formula of MacDonald (*17*). Female *A*. *gambiae* mosquito density (*m*) was estimated to be 3.05 mosquitoes/person/day in the highland forested area, ≈4.64 in the highland deforested area, and ≈8 in the lowland site (*27*). We estimated vectorial capacity to be 77.7% higher in the deforested area than in the forested area in the highlands (Table 2).

**Table 2:** Estimated vectorial capacity of *Anopheles gambiae* mosquitoes in forested and deforested areas, western Kenyan highland and lowland*

Site	Land use type	*m*	*a*	*N*	*P*	Vectorial capacity
Highland	Forested	3.05	0.198	13.9	0.927	0.54
Highland	Deforested	4.64	0.233	12.8	0.917	0.96
Lowland	Deforested	7.85	0.465	11.7	0.923	8.30

*m, relative density of vectors in relation to humans; *a*, average no. children bitten by 1 mosquito in 1 day; *n*, duration of sporogony in days; *P*, proportion of vectors surviving per day. See text for details on data source and assumptions made for calculating each variable.

## Discussion

Environmental changes, whether natural phenomena or the result of human intervention, alter the ecologic context within which vectors and their parasites breed, develop, and transmit disease (*28*). Large-scale conversion of tropical forests for agricultural purposes can change surface properties (e.g., soil wetness, reflectivity, and evaporation rates) of an area, leading to changes in local climate (*29*–*31*). Bounoua et al. (*32*) reported that in the tropics and subtropics, conversion warms canopy temperature by 0.8°C year round. Our study established the role of deforestation on local microclimate and on the sporogonic development of *P*. *falciparum* in *An*. *gambiae* mosquitoes from the western Kenyan highlands. Deforestation appears to alter the climate in highlands, in particular, making it warmer and less humid. Ingestion of *P*. *falciparum* gametocytes by *An*. *gambiae* mosquitoes is more likely to result in infection, and the development time for oocyts and sporozoites is shorter in deforested sites than in forested sites. Given the established effects of temperature on *P*. *falciparum* development, we conclude that changes in climatic conditions caused by deforestation have caused changes in parasite infection rates and development.

Our results are supported by those of other studies. Okech et al. (*33*) showed that in different microhabitats with different temperatures in a lowland site in western Kenya, increasing temperatures led to an increase in infection rates of *An*. *gambiae* mosquitoes fed on blood infected with *P*. *falciparum* gametocytes. However, as with our study, in the study of Okech et al. (*33*), oocyte densities did not differ with increasing temperatures. Noden et al. (*19*) examined the effect of temperature on development of *P*. *falciparum* in *An*. *stephensi* mosquitoes (normally a vector of rodent malaria) and found that the rate of ookinate development was lengthened as temperature decreased from 27°C to 21°C. They concluded that temperature affects sporogonic development of *P*. *falciparum* in anophelines by altering kinetics of ookinete maturation.

Our study has implications for understanding the effects of deforestation on malaria risk in the western Kenyan and other Africa highlands. Force of malaria transmission may be measured by using vectorial capacity. Duration of sporogony of *P*. *falciparum* in mosquitoes is exponentially related to vectorial capacity (*17*). If daily survival and biting frequency of a vector are assumed to be constant, decreasing the duration of sporogony leads to an increase in vectorial capacity. In our study, deforestation led to a decrease in duration of sporogony of *P*. *falciparum* by 1.1 days. Together with other factors that were also influenced by deforestation, such as increased mosquito density, biting frequency, and enhanced survivorship, This decrease translates into an increase in vectorial capacity of *An*. *gambiae* mosquitoes by 77.7%. The implication of this finding is that deforestation in the western Kenyan highlands could potentially increase malaria risk. In African highlands where temperature is an important driving factor for malaria and the human population generally has little functional immunity (*34*), relationships between land use, microclimate, and malaria should be carefully considered during economic development planning to mitigate the effects of malaria on human health.
